# Embolizing Massive Right Atrial Thrombus in a HIV-Infected Patient

**DOI:** 10.1177/2324709618802871

**Published:** 2018-09-28

**Authors:** Piruthiviraj Natarajan, Fowrooz Joolhar, Sudhagar Thangarasu, Ayham Aboeed, Theingi Tiffany Win, Everardo Cobos

**Affiliations:** 1 Kern Medical Center, Bakersfield, CA, USA; 2 2UCLA-David Geffen School of Medicine, Los Angeles, CA, USA

**Keywords:** HIV, right atrial thrombus, embolization, patent foramen ovale, thromboembolism, pulmonary embolus

## Abstract

The risk of thromboembolism is increased when associated with the human immunodeficiency viral (HIV) infection. Various factors are involved in promoting thrombosis, and the presence of a patent foramen ovale augments the potential for a paradoxical embolism. We describe the case of a 56-year-old man receiving antiretroviral therapy with features of right heart failure and pulmonary embolism. Due to the high incidence of life-threatening thromboembolism in the HIV-infected group, the need for long-term anticoagulation has to be evaluated.

## Introduction

The thrombotic risk is increased by 40% in people infected with human immunodeficiency virus (HIV).^1^ The mortality risk with venous thromboembolism among the HIV-infected is high.^2^ Abnormalities in various clotting factors and mechanisms can result in a prothrombotic milieu.^3^ Antiretroviral protease inhibitors were recognized to possibly affect the thrombotic factors synthesized in liver promoting thrombosis.^4^ Opportunistic infections like cytomegalovirus are associated with endothelial damage resulting in a hypercoagulable state.^5^

A patent foramen ovale (PFO) increases the risk of recurrent stroke, and the closure of the atrial septal defect is effective in reducing the stroke risk.^6^ The mortality rate of pulmonary embolism associated with right atrial (RA) thrombus is 44.7%.^7^ Surgical clot removal is reserved for eligible hemodynamically stable patients. The higher risk groups are managed better conservatively, considering the risks associated with the surgical procedure.^8,9^ Recently, ultrasound-accelerated rheolytic thrombectomy for impending paradoxical embolism successfully reduced the complications of thrombosis.^10,11^

## Case Presentation

A 56-year-old man, HIV serology positive for 8 years, presented to the emergency department with progressive worsening of shortness of breath for 2 days. He experienced shortness of breath for the past 6 months. He had bilateral leg swelling and orthopnea in the recent months. The latest cluster definition (CD4) cells count was 804 cells/µL, and he received antiretroviral therapy Genvoya (elvitegravir/cobicistat/emtricitabine/tenofovir alafenamide). He was previously diagnosed with asthma and positive IgG (immunoglobulin G) serology for hepatitis C virus (HCV). He smoked a pack of cigarettes for 20 years and engaged in unprotected sexual acts with men.

On examination, the patient had jugular venous distension and tachypnoea with bilateral basal crackles heard on auscultation. He had bilateral pitting pedal edema (grade 2) reaching the bilateral tibial tuberosity.

**Figure 1.**
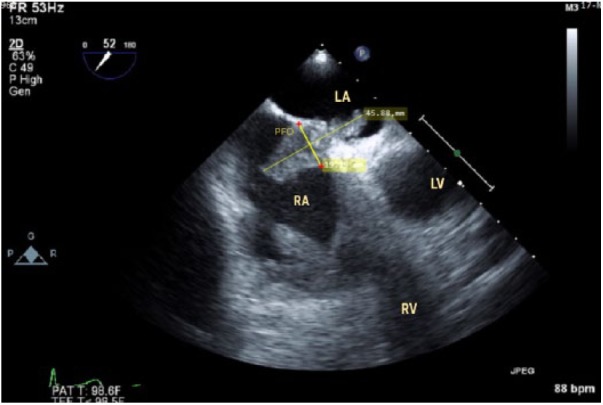
Interatrial thrombus at the time of admission.

**Figure 2.**
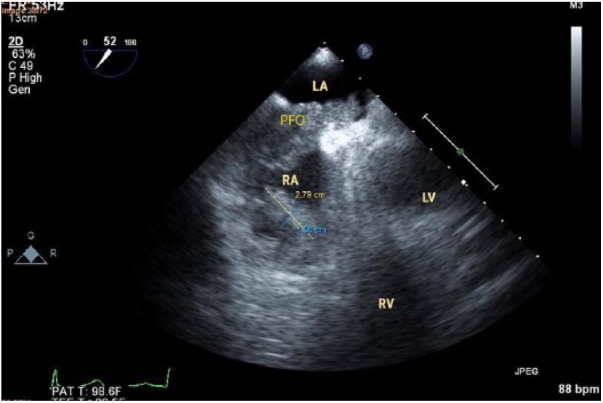
Interatrial thrombus reduced in size after embolization.

**Figure 3.**
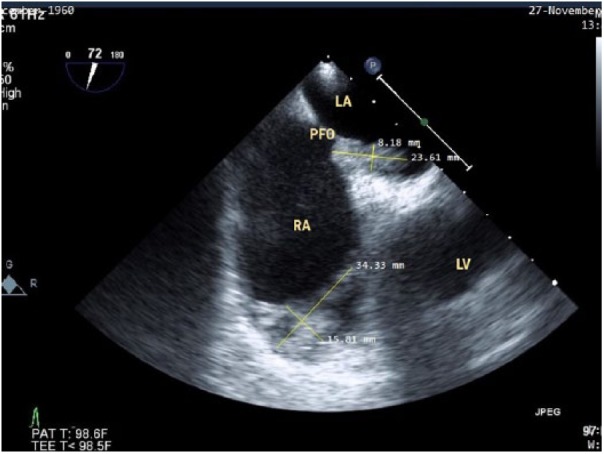
Thrombus tethered to the right tricuspid valve.

His respiratory symptoms worsened despite the immediate resuscitation efforts at the emergency department, and he required a mechanical ventilator due to impending type 1 respiratory failure. The CD4 cell count at the time of admission was 467 cells/µL with the serum HIV-1 viral load of less than 20 copies/mL. The serum HCV RNA viral load by polymerase chain reaction assay was less than 15 IU/mL. Plain chest X-ray showed cardiomegaly and moderate diffuse pulmonary congestion. The brain natriuretic peptide level was 574 pg/mL on admission. The initial transesophageal echocardiogram showed signs of a dilated right ventricle, elevated pressures, and 2 large echodensities with one tethered to the PFO (Figure 1) and another to the tricuspid valve (Figure 2), which suggested RA thrombus. The left ventricular ejection fraction was around 60% with grade 1 diastolic dysfunction associated with a compromised left ventricular size due to the enlarged right ventricle. The interventricular septum showed dyskinesia, secondary to elevated right ventricular pressure and volume. A large complex thrombus with mobile lobulations was found attached to the base of the tricuspid valve with 27.9 × 10.8 mm dimensions. The second thrombus, with complex features, measuring 45.8 × 19.1 mm, had crossed the PFO and protruded into the left atrium. The protruded freely mobile, small linear segment of the echo density measured 10 × 3 mm on the left atrial side. He received low-molecular-weight heparin (enoxaparin) 1 mg/kg to prevent further thrombosis. The qualitative cardiac troponin-I enzyme report was negative. Computed tomography of pulmonary angiogram revealed an eccentric nonocclusive thrombus in the proximal left lower lobar artery and bilateral embolization in the segmental arteries associated with consolidation in the left lower lobe with minimal pleural effusion. Venous ultrasonogram showed no deep vein thrombosis. Transesophageal echocardiogram on the 13th day of admission showed a decreased size of the RA thrombus with features suggestive of ruptured thrombus and distal embolization (Figure 3). The RA thrombus tethered to the tricuspid valve of 3.4 × 1.5 mm size and PFO thrombus of the size 8.1 × 3.1 mm were found to be reduced in size. During the hospital course there was no clinical evidence of any further recognizable embolization. Further hospital stay for the patient was notable for continued intensive care, tracheostomy, and percutaneous endoscopic gastrostomy procedures. Goals of care were discussed with the family, and he was transitioned from critical care to long-term care facility.

## Discussion

The origin of a RA thrombus is usually attributed to the embolization of a thrombus from the deep venous system.^12^ A primary RA thrombus formation can occur with low blood emptying velocity within the appendage due to an increase in RA size.^12^ Iatrogenic causes of RA thrombus formation, including pacemaker leads, indwelling catheters, and mechanical valves, were previously reported.^13,14^ HIV infection, obesity, age, smoking, heart failure, and sedentary life style were risk factors associated with thromboembolism in our patient.^15^ There is a 10-time increase in the incidence rate of thrombotic events associated with HIV infection.^16,17^ Even though an elevated brain natriuretic peptide level and presenting symptoms were suggestive of left heart failure, the etiology of the current presentation could be attributed predominantly only to the thrombus. Acquired protein C and S deficiency, deficient heparin cofactor II, antithrombin deficiency, disorder of plasmin, and increased levels of proinflammatory cytokines innate to HIV infection are the factors commonly promoting thrombosis.^4^ The HCV infection with varying viral loads are independently implicated with prothrombotic effects of higher factor VIII and lower protein S level.^18^ Protease inhibitors are more often associated with prothrombotic effects when the CD4 count is less than 200 or in the presence of AIDS-defining illnesses.^19^ HIV proteases are aspartyl proteases and inhibition of this enzyme result in decreased regulation of coagulation.^20,21^ Moreover, the lipophilic protease inhibitors are metabolized by the cytochrome P450 systems for biotransformation and they indirectly affect cholesterol metabolism. They are speculated to affect hepatic regulation of thrombotic proteins.^22^

The RA thrombus can embolize to the lung and subsequently result in the pressure to rise in the pulmonary system. As the RA pressure rises, the risk of the clot in the RA embolizing through the PFO to the left atrium increases.^23^ This paradoxical embolus can result in a stroke or other end organ damage.^24,25^ Cardiac thrombectomy and PFO closure are preferred for low-risk and hemodynamically stable patients. Patients with higher risk and/or unstable hemodynamics managed with thrombolysis or anticoagulation had better outcomes.^26^

The factors influencing the risk of thromboembolism are often irreversible in HIV-infected patients and a long-term anticoagulation can be useful to prevent further events. Intravenous anticoagulation was provided for our patient. New oral anticoagulant dabigatran administered for 1 year successfully prevented thromboembolic events without any noticeable interaction with the antiretroviral therapy in other case reports.^27^ Current methodology for predicting thromboembolic risk in HIV-infected patients are not clear, and further studies are warranted to evaluate the role of long-term anticoagulation for this subset of patients.

## Conclusion

Thromboembolism in a HIV-infected patient is a potential life-threatening event. Identifying the risk factors for thromboembolism among the HIV-infected individuals and measures for managing with long-term anticoagulation is vital. Further prospective studies to evaluate a new risk score for anticoagulation in this subset is needed.
